# Simultaneous Detection of Multiple Adulterants in Cocoa Powder Using Fourier Transform Infrared Spectroscopy and Chemometrics

**DOI:** 10.1021/acsomega.6c00190

**Published:** 2026-08-20

**Authors:** Amy M. Jackson, Sophie J. Hodge, John R. Bows, Geoff R. Nash

**Affiliations:** † Natural Sciences, Faculty of Environment, Science and Economy, 3286 University of Exeter, Exeter EX4 4QF, U.K.; ‡ PepsiCo R&D, Leicester LE4 1ET, U.K.

## Abstract

Economically motivated adulteration of cocoa powder poses significant risks to food safety, product quality, and consumer trust. Traditional detection methods, while accurate, are often time-consuming and resource intensive. This study presents a novel approach for the simultaneous detection and quantification of carob flour and cocoa shell adulterants in cocoa powder using Fourier transform infrared (FTIR) spectroscopy coupled with partial least-squares regression (PLSR) modeling. More than 600 spectra from 153 samples, including pure, single-adulterant, and mixed-adulterant cocoa powders, were analyzed. A three-stage model development strategy was employed, and the best model, using standard normal variate (SNV) transformation combined with Savitzky–Golay smoothing, achieved high predictive accuracy (CV-*R* ^2^ = 0.946 ± 0.011) with low detection limits (2.28% for carob and 5.80% for shell). Variable importance in projection (VIP) analysis identified key spectral regions contributing to adulterant discrimination. This methodology demonstrates strong potential for scalable, nondestructive quality control in the cocoa industry and offers a framework for multiadulterant detection in other complex food matrices.

## Graphical abstract

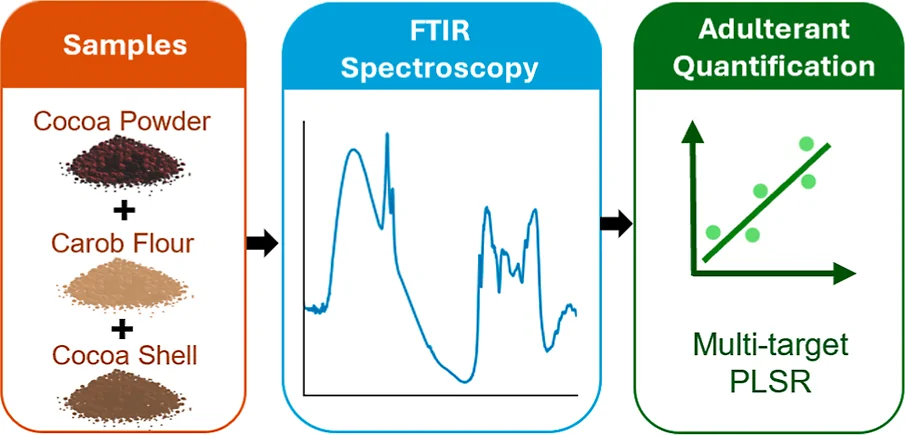

## 1 Introduction

Cocoa powder is a widely utilized ingredient in the food and beverage industry, valued for its distinctive flavor, deep color, and perceived health benefits including antioxidant properties.^1^ Its versatility has driven its incorporation into functional foods and beverages, reflecting a growing consumer demand for healthier, naturally derived products. Cocoa powder production involves a multistep process: harvesting, fermenting, cleaning, drying, roasting and peeling of cocoa beans, followed by the extraction of cocoa butter and grinding of the remaining solids.^2^ Each stage of production can influence the chemical composition and purity of the final product, so effective process control is critical to ensuring product quality.

Over the past decade, the global cocoa powder market has expanded significantly, driven by a rising demand across confectionery, bakery and health food sectors. Supply, however, has struggled to keep pace. Cocoa is predominantly cultivated by smallholder farmers in equatorial regions, with West Africa, particularly Côte d’Ivoire and Ghana, accounting for nearly two-thirds of global production.^3^ Climate related stressors, alongside geopolitical instability and economic pressures have increasingly disrupted cocoa supply.^4^ International Cocoa Organization (ICCO) data indicates that global cocoa production and grindings have fluctuated greatly in recent years, with a notable deficit of 441 thousand tonnes in 2023/24.^5^ Although cocoa prices have fallen in recent months, ICCO reports from mid-2025 indicate that structural challenges and weather variability persist.^6–8^ These recurring supply and economic pressures may incentivise economically motivated adulteration.

Adulteration describes the deliberate substitution of ingredients with low-cost alternatives. This malpractice misleads consumers and can compromise food safety. In cocoa powder, common adulterants include cocoa shells, nutshells, carob, chicory and soy flour.^9^ Roasted carob, in particular, closely mimics cocoa’s sensory profile, making it a profitable substitute that can evade detection. Cocoa shell adulteration may result from ineffective peeling during processing or intentional addition to increase bulk. While complete shell removal is technologically challenging, international food standards prohibit shell content above 5%, with anything above this limit indicative of fraud or poor process control.^10^ Elevated shell content poses several risks, shells may introduce hazardous substances such as heavy metals^11^ and their abrasive fibers can damage processing equipment.^11^ Sensory quality is also affected, with off-flavours and sandy mouthfeel reported in adulterated products.^12^ Therefore, detection of adulterants in cocoa powder is critical for maintaining consumer trust, ensuring product safety and quality, and preserving equipment longevity.

Chromatographic techniques have long been considered the gold standard for adulteration detection due to their sensitivity and specificity. However, they are time-consuming, costly and require skilled personnel. Vibrational spectroscopy techniques like mid-infrared (MIR) and near-infrared (NIR) coupled with multivariate modeling could provide a rapid, cost-effective, nondestructive and scalable alternative. These techniques harness the inherent vibrational modes of molecules to measure absorbance or transmittance in the near- and mid-infrared regions of the electromagnetic spectrum. While NIR spectroscopy is widely adopted for its deeper sample penetration and compatibility with portable devices, MIR spectroscopy offers higher chemical specificity, making it particularly suitable for complex matrices like cocoa powder.

Chemometric modeling plays a pivotal role in interpreting complex spectral data. Among various algorithms, partial least-squares regression (PLSR) is widely favored for its ability to handle collinear and noisy data, making it suitable for quantifying adulterants in heterogeneous food matrices.^13^ Other techniques such as principal component analysis (PCA), support vector machines (SVM), and artificial neural networks (ANN) have also been explored for classification and regression tasks in food authentication.^14,15^ While PCA is effective for exploratory data analysis and pattern recognition, supervised methods like PLSR and SVM often offer superior performance in predictive modeling, especially when dealing with multiple adulterants.

Recent advances in food authentication have demonstrated the effectiveness of vibrational spectroscopy techniques for the detection of adulterants in powdered food matrices.^16,17^ NIR based approaches are particularly well established and have been widely applied across diverse food systems, including spices, cereals and dietary supplements, due to their rapid, nondestructive nature and ability to achieve high classification and quantification accuracy when coupled with multivariate techniques.^13^ In cocoa systems specifically, infrared spectroscopy, combined with multivariate analysis, has proven effective in detecting adulterants including carob flour and cocoa shell at levels below 5%.^18–21^ More recently, Millatina et al.^22^ extended NIR approaches to cover more adulterant types including foxtail millet, soybean and wheat flour, however, these were evaluated as single-adulterant systems in model calibration. Existing studies on cocoa powder are limited to simple, binary adulteration scenarios so their practical applicability remains limited. Simultaneous detection of multiple adulterants introduces unique analytical challenges. Spectral overlap between different adulterants can obscure individual signatures, complicating model calibration and interpretation and reinforcing the need for chemometric analysis.

Beyond cocoa powder, recent advances in multiadulterant detection have shown that infrared spectroscopy combined with chemometric modeling can effectively identify multiple trace adulterants in complex food matrices. For example, multimolecular infrared (MM-IR) spectroscopy integrated with PLS modeling has achieved simultaneous detection of 5 adulterants in flour with high sensitivity and without the need for sample separation.^23^ Similarly, NIR spectroscopy combined with chemometrics has successfully quantified multiple adulterants in goat milk powder.^24^ A related study on roasted coffee demonstrated the ability to discriminate between pure and adulterated samples with up to six adulterants by combining NIR and PCA, although accurate identification was limited to single or dual adulterations above 10%.^25^ Comparable strategies have also been applied to liquid systems, for instance, Fourier transform infrared spectroscopy (FTIR) combined with PLSR has achieved excellent predictive accuracy for binary and ternary edible oil mixtures,^26^ further demonstrating the effectiveness of multivariate analysis in complex food applications.

Despite this progress, a clear methodological gap remains in cocoa powder authentication. To date, there are no studies that demonstrate simultaneous quantification of multiple adulterants in cocoa powder using MIR spectroscopy. Furthermore, most prior cocoa focused studies rely on attenuated total reflectance (ATR) or diffuse reflectance based NIR measurements, while application of FTIR with KBr pellet preparation has not yet been systematically explored for this purpose. The use of KBr pellets can further enhance MIR analysis by improving spectral clarity and reducing baseline distortion, facilitating more accurate interpretation of subtle chemical differences in complex powdered matrices. In this work we address these limitations by presenting a novel approach to cocoa powder quality control through the development of a multiadulterant detection framework using KBr pellets, FTIR spectroscopy and PLSR modeling. By explicitly modeling mixtures containing multiple adulterants, this study extends beyond binary systems to better reflect realistic fraud scenarios. This method therefore offers a scalable, industry-relevant solution for safeguarding product integrity, ensuring regulatory compliance, and protecting consumer health.

## 2 Materials and Methods

### 2.1 Sample Preparation

Samples were prepared using commercially available ingredients, detailed in Table S1. Cocoa powder (Food Thoughts UK 100% organic cocoa powder) was manually adulterated with carob flour (Whole Food Earth Ltd. organic carob flour) and cocoa shell (from Speedrange Spices Ltd. organic cocoa beans) at concentrations between 0 and 25% (w/w) to simulate realistic contamination scenarios. Prior to mixing, cocoa powder and carob flour were sieved, while cocoa shell was ground and then sieved to ensure uniform particle size and maximize homogeneity. Samples were prepared as standard potassium bromide (KBr) pellets, commonly used in infrared spectroscopy, maintaining a consistent concentration of 5% adulterant mixture. To prepare each sample, 0.285 g of KBr was finely ground for 30 s to minimize moisture uptake, then combined with 0.015 g of the cocoa–adulterant mixture. The resulting blend was stirred until homogeneous and pressed into pellets using a Specac mini-pellet press at 2 tonnes for 10 s.

A total of 17 unique mixtures were prepared including pure cocoa, single-adulterant and multiadulterant samples (full details in Table S2). Nine pellets of each mixture were prepared, and each pellet was measured 4 times, rotating 90° between measurements to account for sample heterogeneity. This resulted in more than 600 spectra from 153 distinct samples with 35% mixed-adulterant samples to support model validation.

### 2.2 FTIR Spectroscopy + Spectral Preprocessing

Transmission spectra of all samples were acquired using a benchtop Shimadzu IRSpirit-X Compact FTIR spectrometer, with measurements undertaken at room temperature under ambient conditions. Each spectrum resulted from an average of 20 scans in the wavenumber region 400–4000 cm^–1^. Each pellet was measured 4 times with 90° rotational increments. Automatic background correction was applied using the feature in Shimadzu’s LabSolutionsIR software. All recorded spectra were converted to absorbance using the relationship *A* = −log_10_(*T*), and subsequent data analysis and modeling were performed using absorbance spectra in accordance with standard chemometric practice.

To optimize model performance, six preprocessing techniques were applied to the absorbance spectra: raw spectra without preprocessing, standard normal variate (SNV) transformation, multiplicative scatter correction (MSC), min–max scaling, Savitzky–Golay (SG) smoothing and a combined SNV and SG smoothing approach. SNV transformation removed multiplicative scatter effects by centring and scaling each spectrum individually, MSC corrected both additive and multiplicative scatter effects by aligning each spectrum to the mean spectrum, min–max scaling rescaled data to normalize feature magnitudes and SG smoothing reduced noise while preserving peak shapes by fitting a polynomial to a moving window across the spectrum. Finally, the combination of SNV and SG smoothing was employed to combine scatter correction and noise reduction thus enhancing signal clarity and consistency across the samples. All data preprocessing was performed using Python within a Jupyter notebook environment embedded in visual studio code.

### 2.3 Model Development: Multi-target Partial Least Squares Regression

A multitarget PLSR framework was implemented to simultaneously quantify carob and shell adulteration in cocoa powder. All models were developed using absorbance-transformed spectra. Model development followed a three-stage optimization approach. In the first stage, preprocessing methods were compared using a fixed number of 10 PLS components and 5-fold cross validation to ensure unbiased selection. In the second stage, the highest performing preprocessing method was subjected to component optimization using the elbow method; up to 15 components were tested and the optimal number was determined based on minimal performance improvement defined as a change in the coefficient of determination *R* ^2^ of below 0.01. In the third stage, nested cross-validation was employed to provide unbiased performance estimates and minimize overfitting during model development. The outer loop used 5-fold stratified cross-validation for model evaluation, while the inner loop used 3-fold cross-validation for hyperparameter tuning and model selection. Stratification was based on total adulterant content to ensure representative fold distribution. By separating model optimization from performance evaluation, this approach ensured that preprocessing selection and latent variable optimization were performed exclusively within the training data of each outer fold, thereby reducing the risk of optimistic bias in estimated model performance. Finally spectroscopic feature interpretation was performed using variable importance in projection (VIP) analysis combined with regression coefficient screening to identify chemically meaningful spectral regions. To support interpretation of the quantitative model, exploratory principal component analysis (PCA) and PLS discriminant analysis (PLS-DA) were performed on the SNV + SG preprocessed spectra using binary labels (adulterated vs nonadulterated) to assess spectral discriminability. Results are presented in Figure S1.

All analyses were conducted using Python 3.12.6, PLS modeling was performed with scikit-learn, data manipulation with NumPy and Pandas, visualization with Matplotlib and Seaborn, and statistical testing with SciPy. Final model validation was carried out using 10-fold cross-validation on the complete data set, employing the optimal preprocessing method (SNV combined with SG smoothing) and 11 PLS components, as determined through the three-stage optimization process. This provided robust performance estimates ensuring model reliability for practical applications.

## 3 Results and Discussion

### 3.1 Model Development and Validation

PCA and PLS-DA analysis confirmed that adulterated and nonadulterated samples are spectrally discriminable (PLS-DA 5-fold CV accuracy = 94.9%, Figure S1), providing qualitative justification for the supervised quantitative modeling approach. A permutation test using 1000 random label permutations confirmed that classification performance was significantly greater than expected by chance (*p* < 0.001).

A three-stage approach was employed to eliminate selection bias in preprocessing method comparison for the development of a multiadulterant model. In the first stage preprocessing comparison using identical hyperparameters (10 PLS components) revealed significant performance differences between methods. While all preprocessing methods resulted in strong model performance with coefficient of determination CV-*R* ^2^ > 0.85 (full results in Table S3), SNV transformation combined with SG smoothing (SNV_smoothed) gave the best performance metrics across the board with CV-*R* ^2^ = 0.930 ± 0.004 and cross validated root mean squared error (CV-RMSE) = 1.93 ± 0.055. This preprocessing method was then selected to progress to stage 2 for component optimization.

In the second stage of model development, 5-fold cross-validation was harnessed alongside “elbow point” analysis to select the optimum number of PLS components by systematically evaluating 1–15 components with SNV_smoothed preprocessing. In this study we define the elbow point as the point at which adding more components results in a <0.01 improvement in CV-*R* ^2^. Marginal improvement per additional component increases the likelihood of model overfitting and could raise concerns over model complexity. The choice of improvement threshold therefore represents a balance between model complexity and performance gains. In Figure 1A CV-*R* ^2^ is plotted as a function of the number of components and shows the elbow point at 11 components, where performance improvement drops below the threshold (Figure 1B).

**Figure 1:**
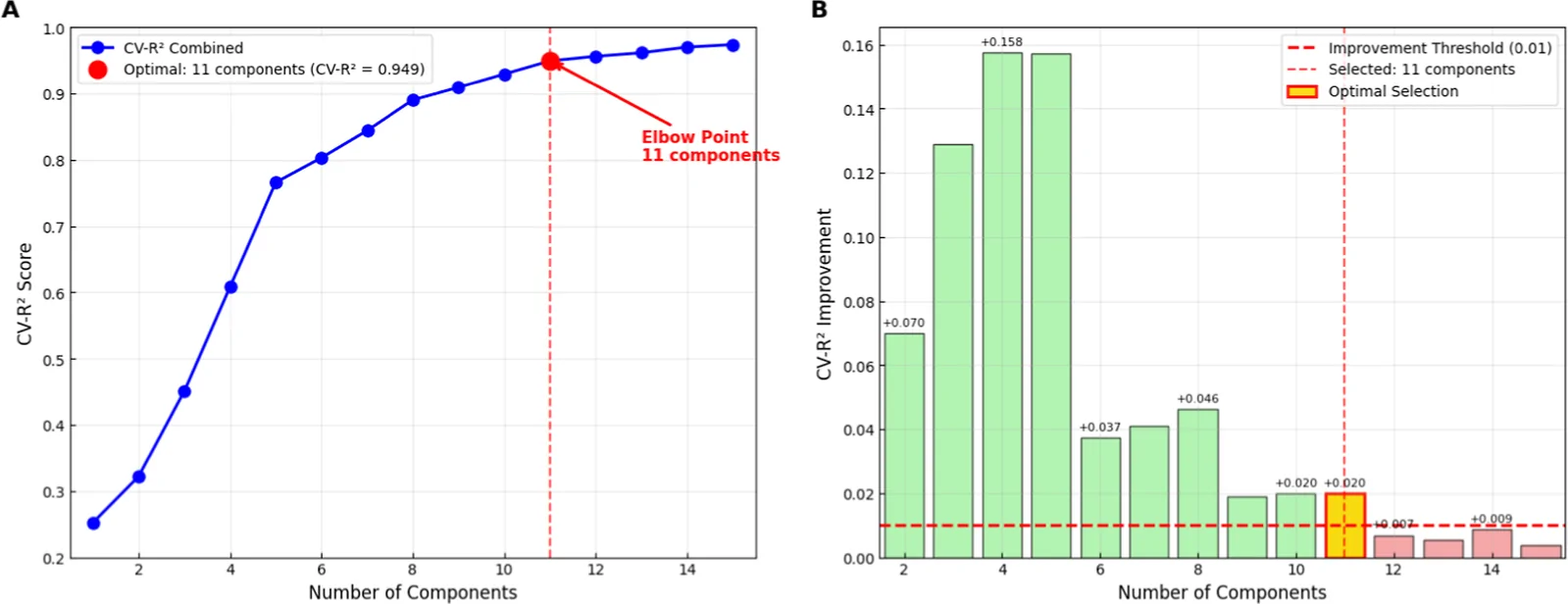
Stage 2 component optimization using elbow method. (A) CV-*R* ^2^ performance up to 15 components. (B) CV-*R* ^2^ improvement per additional component with threshold line (0.01).

In the third stage, to provide unbiased performance estimations, nested cross validation was implemented with 5 outer folds and 3 inner folds (Figure 2). The outer loop provided true performance estimation while the inner loop performed hyperparameter optimization within each training fold to ensure no data leakage between model selection and evaluation phases. Figure 2A confirms that SNV_smoothed achieved the highest mean performance (CV-*R* ^2^ = 0.951 ± 0.004). The low standard deviation (±0.004) indicates robust and consistent performance across validation folds. Similarly, Figure 2B demonstrates that the SNV_smoothed model achieved the lowest prediction error (MAE = 1.27 ± 0.047%), representing a 22% improvement over raw absorbance spectra (MAE = 1.62 ± 0.316%).

**Figure 2:**
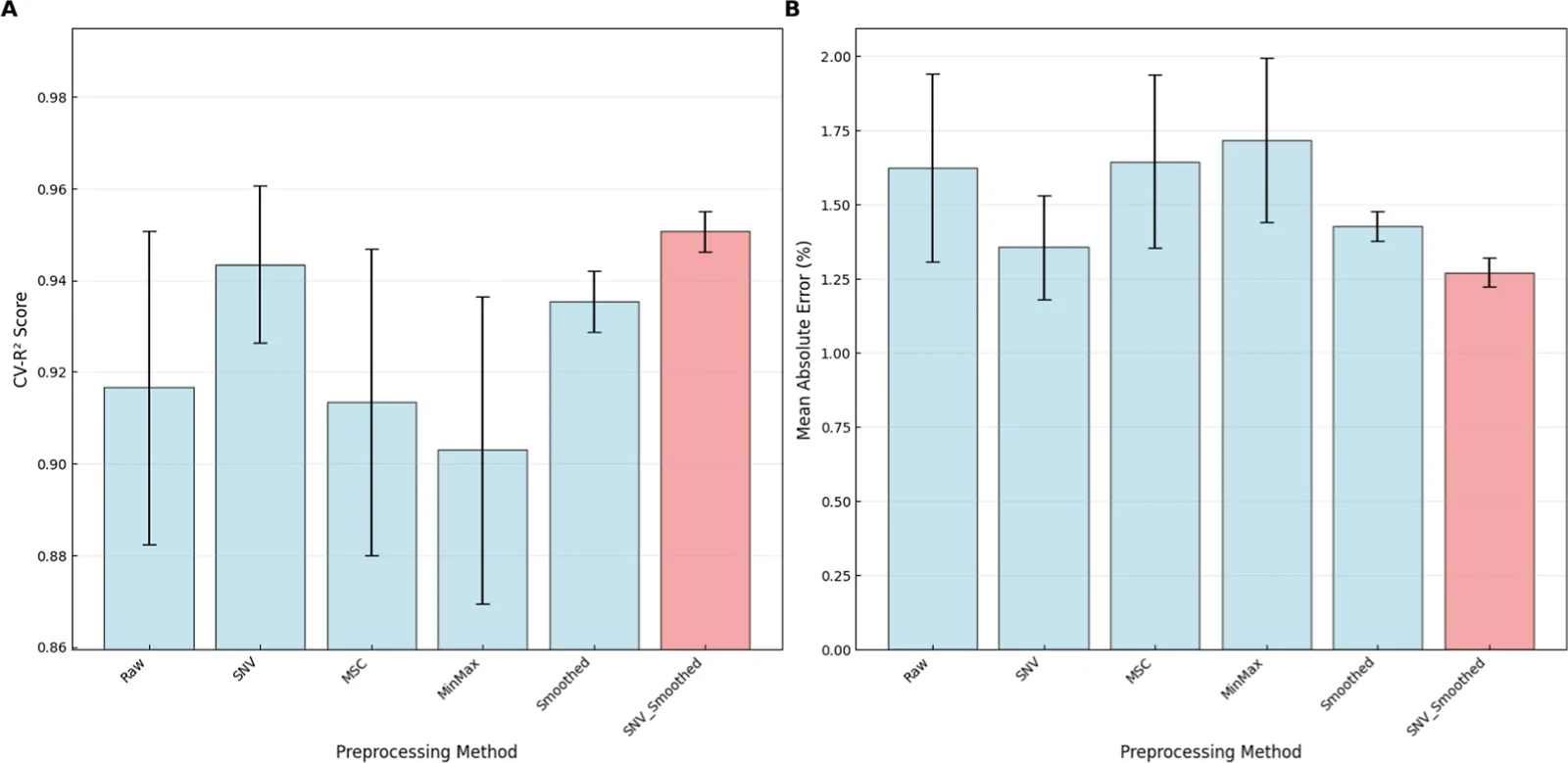
Stage 3 nested cross validation performance. (A) CV-*R* ^2^ scores and (B) mean absolute error (MAE) with standard deviation across 5 folds. The best performing model (SNV_smoothed) is highlighted in red.

Beyond performance estimation, comprehensive pairwise statistical testing was conducted using paired *t* tests with Cohen’s *d* effect size calculations to quantify both statistical significance and practical importance of differences in preprocessing methods. The pairwise comparisons controlled for fold to fold variation in data complexity and demonstrated that preprocessing choice substantially impacts model performance, with 3 of 15 comparisons showing statistical significance (*p* < 0.05). Notably the SNV_smoothed model significantly outperformed the MinMax model (*p* = 0.034, Cohen’s *d* = −2.226) and SG smoothing alone (*p* = 0.003, Cohen’s *d* = −3.023). These large effect sizes (|*d*| > 2.0) indicate that the observed improvements are not only statistically significant but also practically meaningful, again emphasizing the importance of systematic preprocessing selection and underscoring the robustness of the SNV_smoothed method.

### 3.2 Multitarget Prediction Performance

The final optimized model demonstrated excellent performance for simultaneous carob and shell detection with CV-*R* ^2^ = 0.946 ± 0.011 and CV-RMSE = 1.658 ± 0.138. Figure 3A,B illustrate individual adulterant prediction CV-*R* ^2^ values of 0.963 ± 0.012 and 0.929 ± 0.018 for carob and shell content, respectively. The strong correlation between predicted and actual adulterant concentrations across the whole range studied indicates robust quantitative capability. Figure 3C highlights the model’s capability of handling mixed adulterant scenarios which may be more representative of real-world cases. The limit of detection (LOD) for each adulterant type was conservatively defined as 3 standard deviations of the predicted concentrations for blank, unadulterated samples. LODs of 2.28% for carob and 5.80% for shell were achieved, with carob detection operating well below the regulatory threshold and shell detection at the practical decision boundary for cocoa powder authentication.

**Figure 3:**
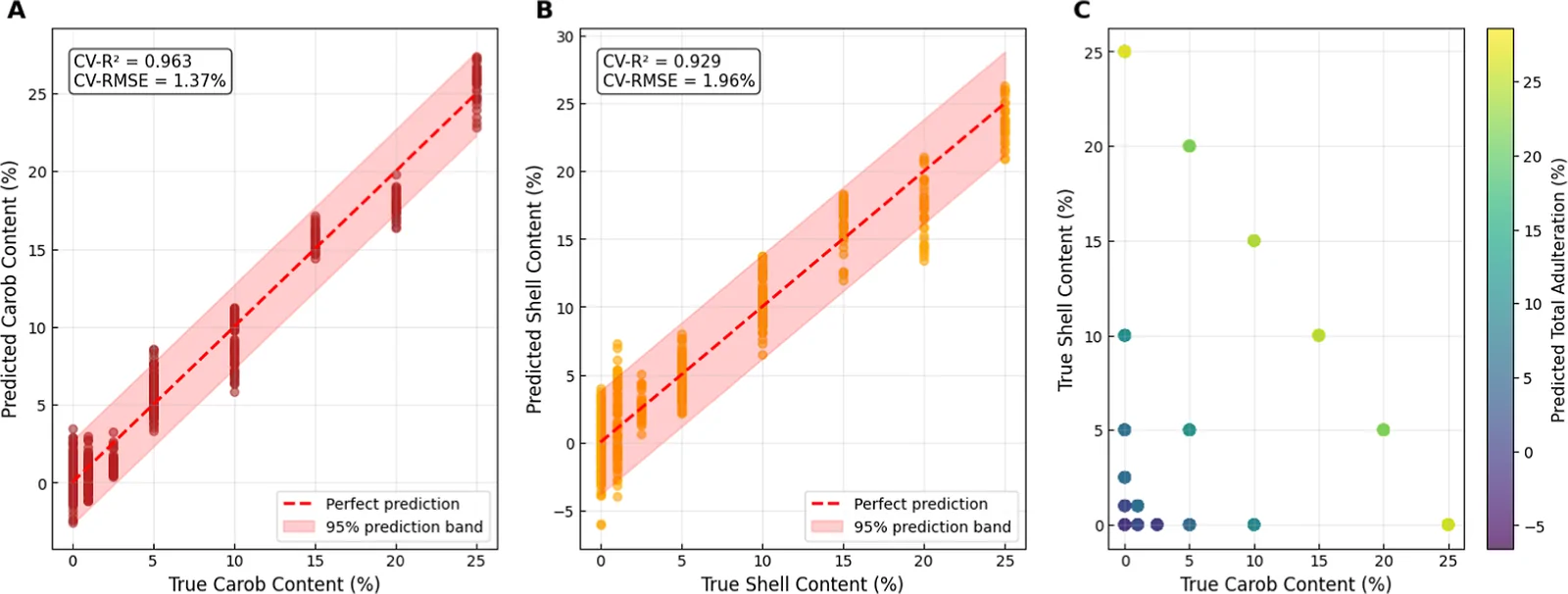
Multi-adulterant detection model performance. (A) Carob and (B) shell content prediction from a single simultaneous multitarget PLSR model; all points are 10-fold cross-validated held-out predictions with 95% prediction bands indicating model uncertainty. (C) Simultaneous adulteration map with color bar indicating total predicted adulteration level. Model calibration performance is reported in Figure S2.

While the model performed well under controlled laboratory conditions, where samples were manually adulterated, validation with industrial samples containing natural compositional variation would strengthen the practical applicability of this approach. Although nested cross-validation was employed to minimize overfitting and provide robust estimates of model performance, validation using independent samples collected from different cocoa batches, operators, instruments, and production environments would further strengthen confidence in model transferability. Future work should therefore prioritize external validation across diverse cocoa origins and processing conditions to assess real-world robustness. Additionally, the model should be extended to include other common adulterants such as wheat flour, corn starch and foxtail millet to provide more comprehensive quality control capabilities. Nonlinear methods (e.g., SVR, neural networks)^13,27^ and modelative classification approaches such as SIMCA^28^ could also be explored.

### 3.3 Spectroscopic Feature Interpretation

Variable importance in projection (VIP) analysis serves as the chemical basis for adulterant discrimination by highlighting the variables that are most influential in explaining the relationship between predictor and response variables. This technique enables evaluation of each part of the spectra’s relative contribution to overall model performance, providing useful insight for interpreting complex spectral data where spectral signals are subtle or overlapping. Variables with VIP scores exceeding 1.0 are considered highly informative for model predictions. In this work VIP scores were harnessed solely for chemical interpretation rather than as a feature-selection step to inform model development, as done in some previous studies.^29^ Given the strong performance of our model, further dimensionality reduction was deemed unnecessary; therefore, the full spectral range was retained to preserve all available spectral information.

To distinguish genuine chemical signals from preprocessing artifacts, we implemented a dual criteria classification requiring both high VIP scores and significant regression coefficients. This strategy mitigates the risk of misinterpreting spectral noise as meaningful features. Figure 4 displays the VIP analysis across the complete infrared spectrum with color coding indicating feature classification. Of 5043 wavenumbers measured, 1165 features (23.1%) exceeded the importance threshold and 53% of these were validated as true chemical signals (green segments), while the remainder were attributed to noise or preprocessing artifacts (red segments).

**Figure 4:**
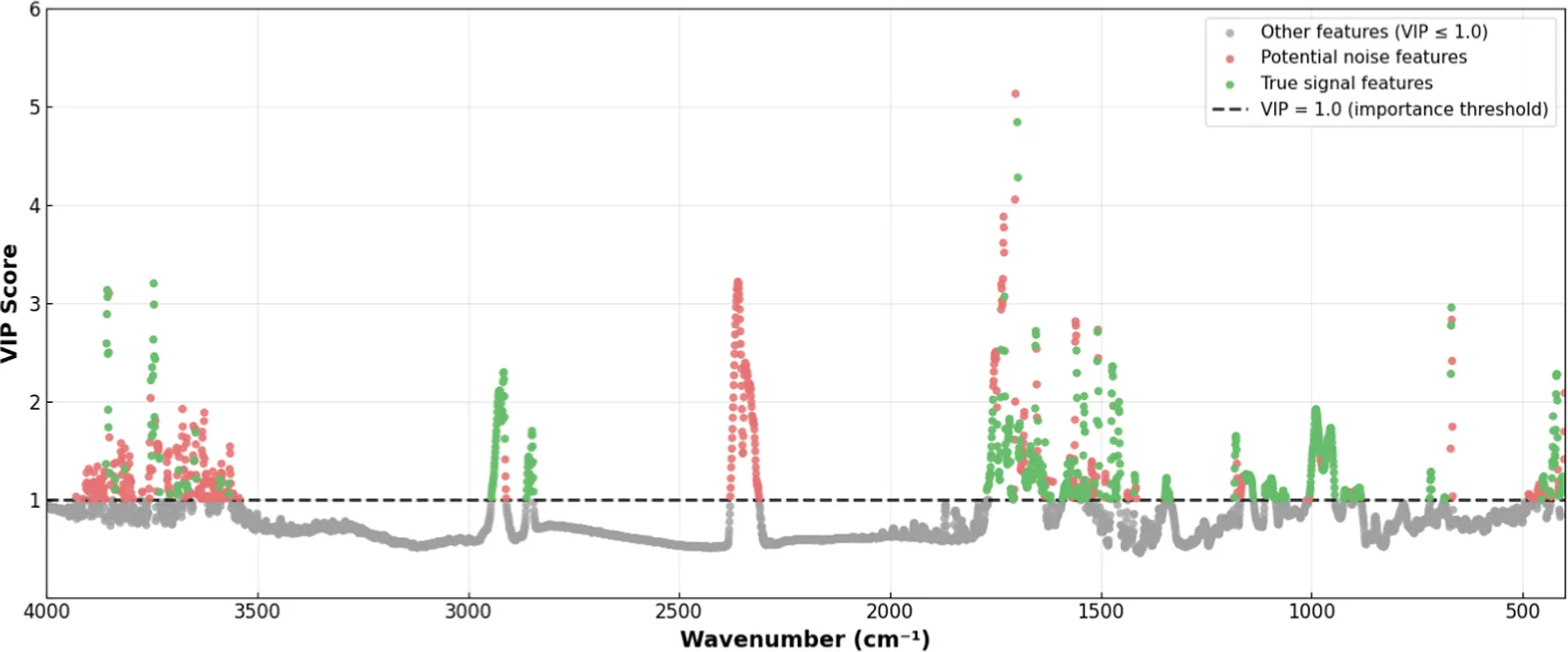
Variable importance in projection (VIP) analysis. Black dashed line indicates conventional importance threshold (VIP = 1) and color shows feature classification. Green segments represent validated spectroscopic signals (high VIP + high regression coefficient) while red segments indicate potential preprocessing artifacts (high VIP + low regression coefficient).

Cocoa powder, carob flour and cocoa shell have several key differences in their biochemical composition. Carob contains higher levels of carbohydrates, natural sugars, and dietary fiber, along with a distinct protein profile and elevated calcium content. In contrast, cocoa powder is richer in fats, proteins, and polyphenolic compounds such as theobromine and caffeine, and has higher potassium levels.^30^ Cocoa shells differ further, exhibiting significantly higher fiber and lower fat content compared to cocoa powder.^31^ These compositional differences in lipid, carbohydrate, protein and polyphenol content align with identified “true signal” regions in Figure 4.

Figure 4 shows that the most discriminatory features were distributed across multiple functional group regions: fingerprint region vibrations (800–1000 cm^–1^), C–O stretch and fingerprint combinations (1000–1200 cm^–1^) and C=O stretch vibrations (1700–1800 cm^–1^). The dependence on CO_2_ absorption (2300–2400 cm^–1^) and water vapor (3600–3700 cm^–1^) reflects the residual changes in atmospheric absorption that were not accounted for by background correction. The greatest “true signal” VIP score (4.839) corresponds to C=O stretch vibrations around 1700 cm^–1^, arising from lipid and protein carbonyl groups, which differ significantly between cocoa, carob, and shell materials. Other discriminatory features included aromatic C=C and N–H vibrations (1400–1600 cm^–1^), aliphatic C–H stretches (2800–3000 cm^–1^), and low-frequency skeletal vibrations (400–500 cm^–1^), all contributing to the model’s ability to differentiate between authentic and adulterated samples. The broad distribution of validated features across the spectrum illustrates the model’s reliance on multidimensional signals rather than isolated chemical markers. This enhances the robustness of model performance and reduces the risk of error.

## 4 Conclusions

A combination of Fourier transform infrared spectroscopy and chemometrics was used for the detection of both carob flour and cocoa shell in cocoa powder. The model achieved strong overall performance metrics with CV-*R* ^2^ = 0.946 ± 0.011 and CV-RMSE = 1.658 ± 0.138. Low detection limits were also achieved at 2.28% and 5.80% for carob and shell, respectively. The model performance is broadly comparable to previous single-adulterant studies, while providing the added advantage of simultaneous multiadulterant detection. The three-stage model development approach highlighted the requirement for appropriate preprocessing conditions and hyperparameter optimization. These results demonstrate the feasibility of developing a methodology for multiadulterant detection and could be readily applied to authentication of a wide variety of complex food matrices, supporting broader efforts in food authentication and fraud prevention.

## Supplementary Material



## Notes

Material specifications (Table S1); mixture design matrix (Table S2); PCA and PLS-DA score plots (Figure S1); stage 1 preprocessing comparison (Table S3); and calibration and cross-validation performances (Figure S2) (PDF)
