# Supplementary material for: Simultaneous Detection of Multiple Adulterants in Cocoa Powder Using Fourier Transform Infrared Spectroscopy and Chemometrics
Source: ACS Omega. 2026 Aug 20;11(34):50762–8. doi: 10.1021/acsomega.6c00190 (PMC13625114; doi:10.1021/acsomega.6c00190)
Supplement: Supplementary file 1 [file ao6c00190_si_001.pdf]

SUPPORTING INFORMATION

Simultaneous Detection of Multiple Adulterants in Cocoa Powder using Fourier Transform Infrared Spectroscopy and Chemometrics.

Amy M. Jackson[1*], Sophie J. Hodge[1], John R. Bows[2] and Geoff R. Nash[1]

[1]Natural Sciences, Faculty of Environment, Science and Economy, University of Exeter, EX4 4QF, United Kingdom
[2]PepsiCo R&D, Leicester, LE4 1ET, United Kingdom

*Corresponding author: aj675@exeter.ac.uk

Table S1. Material specifications and traceability information for all source materials used in sample preparation.

Material	Supplier	Specification	Batch Number	Origin	Best Before
Cocoa Powder	Food Thoughts UK	100% Organic Cocoa Powder	4276	Single Source (unknown)	April 2026
Carob Flour	Whole Good Earth Ltd	Organic Carob Flour	32209	Italy	November 2025
Cocoa Shell	Speedrange Spices Ltd	Organic Cocoa Beans	141A406	Peru	June 2026

Table S2. Design matrix of the 17 unique mixtures used for model development, detailing the percentage composition of carob flour, cocoa shell, and cocoa powder, and the number of replicates per mixture.

Mixture	Carob (%)	Cocoa shell (%)	Cocoa (%)	Replicates
1	0	0	100	36
2	0	1	99	36
3	0	2.5	97.5	36
4	0	5	95	35
5	0	10	90	36
6	0	25	75	36
7	1	0	99	36
8	1	1	98	36
9	2.5	0	97.5	36
10	5	0	95	36
11	5	5	90	36
12	5	20	75	36
13	10	0	90	36
14	10	15	75	36
15	15	10	75	36
16	20	5	75	36
17	25	0	75	36

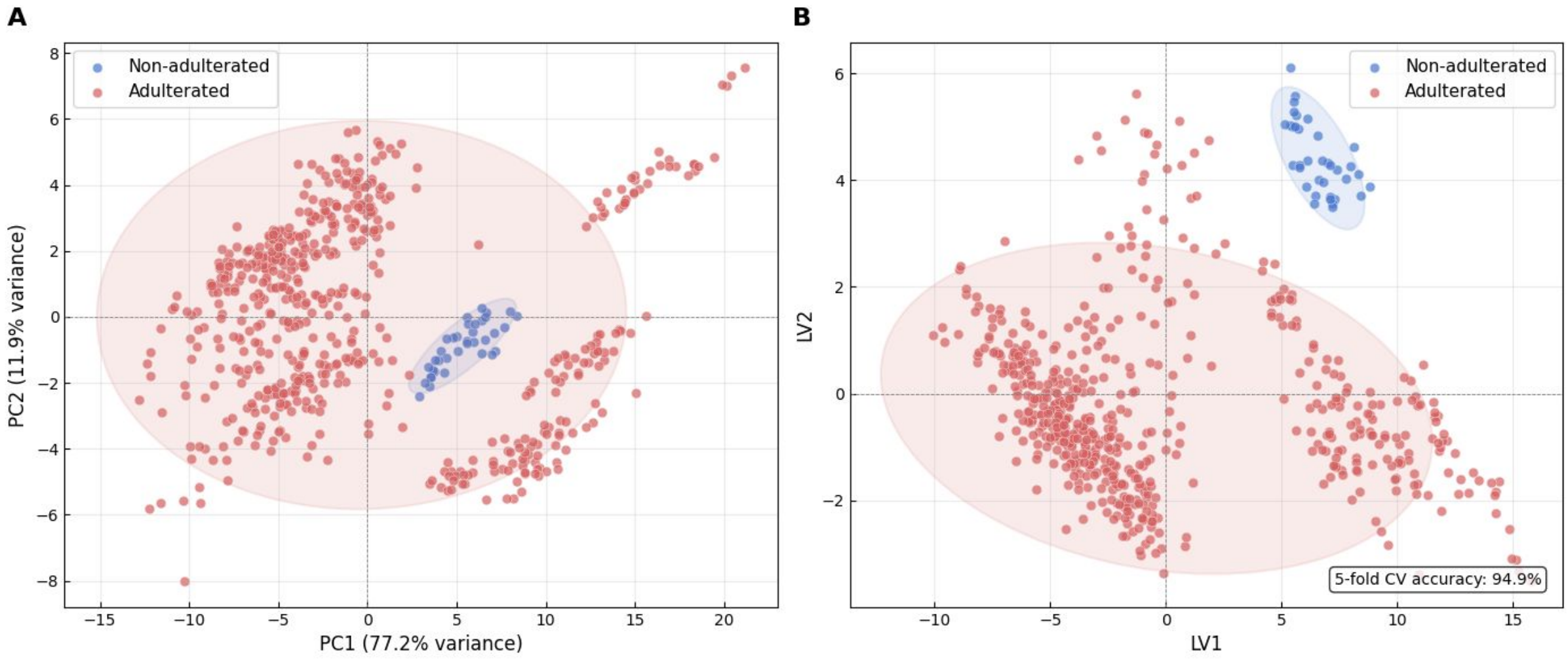

Figure S1. Unsupervised and supervised discrimination of adulterated and non-adulterated cocoa powder samples using SNV+SG preprocessed spectra. (A) PCA score plot (PC1 vs PC2); PC1 captures 77.2% of total spectral variance. (B) PLS-DA score plot (LV1 vs LV2). Classification performance was assessed using 5-fold stratified cross-validation with out-of-fold predictions, yielding a cross-validated classification accuracy of 94.9%, calculated as the proportion of correctly classified samples. Robustness to chance correlation was evaluated using a permutation test ($n = 1000$), which confirmed that the observed classification performance was significantly greater than expected by chance ($p < 0.001$). Blue: non-adulterated; red: adulterated samples.

Table S3. Stage 1 preprocessing comparison. All six methods evaluated using 5-fold cross-validation with fixed n = 10 PLS components. CV-R^2 = cross-validated coefficient of determination; CV-RMSE = cross-validated root mean squared error (%). Methods ranked by combined CV-R^2.

**Rank**	**Preprocessing Method**	**CV-R^2**	**CV-R^2 Std Dev**	**CV-RMSE Combined**	**CV-RMSE Std Dev**
1	SNV_Smoothed	0.9292	±0.0040	1.929	±0.055
2	SNV	0.9222	±0.0050	2.047	±0.063
3	SG Smoothed	0.9020	±0.0123	2.219	±0.085
4	Raw	0.8978	±0.0073	2.332	±0.085
5	MSC	0.8884	±0.0113	2.390	±0.105
6	Min-max	0.8503	±0.0268	2.823	±0.228

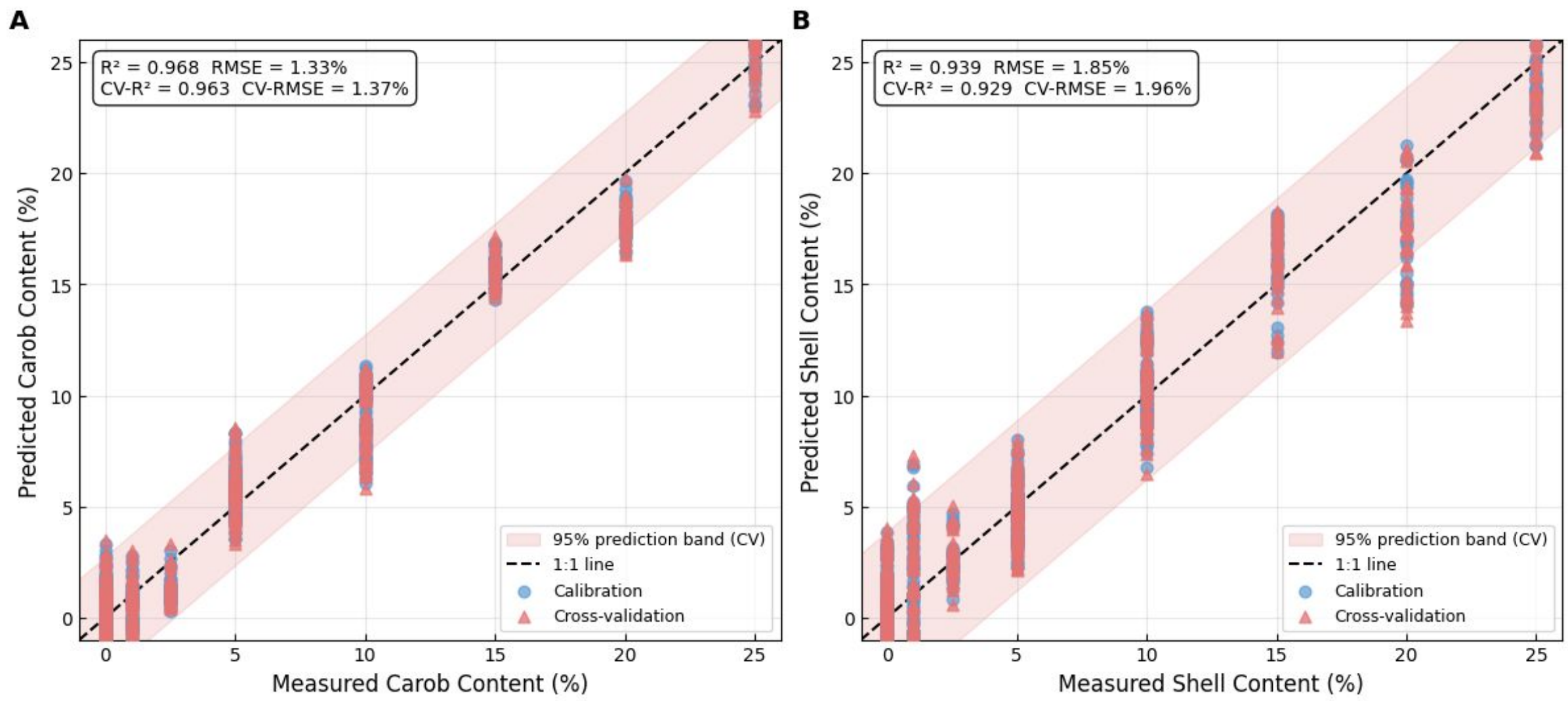

Figure S2. Calibration and cross-validation prediction performance for (A) carob and (B) shell content. Blue circles: calibration predictions (R^2); red triangles: 10-fold cross-validated predictions (CV-R^2). The negligible gap between R^2 and CV-R^2 gap (0.005 carob, 0.010 shell) confirms model validity without overfitting.
